# Supplementary material for: Conspicuous colours reduce predation rates in fossorial uropeltid snakes
Source: PeerJ. 2019 Aug 14;7:e7508. doi: 10.7717/peerj.7508 (PMC6698130; doi:10.7717/peerj.7508)
Supplement: Supplemental Information 1 [file peerj-07-7508-s001.docx]

**Supplemental Methods S1.**

*Model design for the field experiment*

For the field experiment, we used clay snake models painted with different coloured acrylic paints to examine the effect of colour on avian predation rates in BBTC tea plantations. Models were made of brown pre-coloured clay (Play clay, Uday Industries^TM^). Brown clay was rolled by hand into a cylinder with one tapering end representing the head and the other end cut obliquely to represent the tail of a uropeltid snake. A twisted metal wire having a loop at one end was embedded into each model, protruding out 3 cm from the tapering end. Each model had a length of ca. 25cm (excluding the protruding metal wire) and a net weight of ca. 18 gm. The models were bent into an ‘S’ shape representing the general posture of a snake before being painted over with acrylic paint (Camel Fabrica^TM^). We replicated the yellow and red paints to match the colour of the two species of uropeltid snakes. We obtained the yellow and red colours by mixing different acrylic paints and modeling the ability of birds to differentiate each colour combination from the snake’s spectral reflectance. We modeled the ability of birds to distinguish the colours using the Receptor Noise Limited (RNL) model (Vorobyev and Osorio, 1998) in the R package Pavo v. 2.1.0 (Maia et al., 2019). We used the relative proportion of cone types of 1:2:4:4 for domestic chickens (Kram et al., 2010) representing the VS visual system of birds along with a Waber fraction of 0.06 (Olsson et al., 2015) modeled under forest shade illumination to model the colours. We chose the colour combinations that had the lowest ‘Just noticeable distance’ (JND) compared to the snake colours (supplementary material, Table S1). The final combinations were a mixture of lemon yellow (236), Vandyke brown (446), sap green (391) and white (478) in the ratio 1.4: 0.1: 0.1: 0.1 respectively for the *U. liura* yellow, and a mixture of scarlet (393), lemon yellow (236) and white (478) in the ratio 0.3: 0.3: 1.2 respectively for the *T. cf. sanguineus* red. The novel colour was a mixture of the *U. liura* yellow and *T. cf. sanguineus* red in 1:1 ratio. We used Vandyke brown and black for the controls. For each of the brightly coloured treatments (red, yellow and novel), we first painted the models with black throughout and then painted the yellow, red and novel colours on the ventral side.

**References**

Kram, Y.A., Mantey, S. and Corbo, J.C., 2010. Avian cone photoreceptors tile the retina as five independent, self-organizing mosaics. PloS one, 5(2), p.e8992.

Maia, R., Gruson, H., Endler, J.A. and White, T.E., 2019. pavo 2: new tools for the spectral and spatial analysis of colour in R. Methods in Ecology and Evolution 0. DOI: 10.1111/2041-210X.13174.

Vorobyev, M. and Osorio, D., 1998. Receptor noise as a determinant of colour thresholds. Proceedings of the Royal Society of London B: Biological Sciences, 265(1394), pp.351-358.
