# Supplementary material for: Conspicuous colours reduce predation rates in fossorial uropeltid snakes
Source: PeerJ. 2019 Aug 14;7:e7508. doi: 10.7717/peerj.7508 (PMC6698130; doi:10.7717/peerj.7508)
Supplement: Supplemental Information 2 — ΔS Values >3.00 are considered as easily distinguishable, values between 1.00 and 3.00 are distinguishable only under optimal light conditions and values <1.00 are indistinguishable from the background. [file peerj-07-7508-s002.docx]

**Supplemental Table S1.**

**Table S1:** Modeling the conspicuousness of the five treatments (clay snake models) in the field experiment against the ventral colouration of the two species of uropeltid snakes (*Uropeltis liura* and *Teretrurus cf. sanguineus*) and the leaf litter background from the four transects (Kakachi 1, Kakachi-2, Cullinia, Manjolai) in BBTC. ΔS Values > 3.00 are considered as easily distinguishable, values between 1.00 and 3.00 are distinguishable only under optimal light conditions and values < 1.00 are indistinguishable from the background.

| **Treatments** | ***U. liura*** | ***T. cf. sanguineus*** | **Kakachi–1** | **Kakachi–2** | **Cullinia** | **Manjolai** | **Leaf average** |
| --- | --- | --- | --- | --- | --- | --- | --- |
| Yellow | 0.5195 | 3.9639 | 5.2441 | 5.3865 | 4.3672 | 4.0235 | 4.6170 |
| Red | 4.3690 | 0.7666 | 3.7940 | 4.3968 | 3.2030 | 3.4454 | 3.5782 |
| Novel | 3.3446 | 1.4134 | 2.6997 | 3.1935 | 1.8808 | 1.9645 | 2.2784 |
| Black | 4.9152 | 6.5616 | 9.7951 | 10.1322 | 8.8950 | 8.7206 | 9.2435 |
| Brown | 7.5867 | 4.0506 | 4.5935 | 5.1787 | 4.6900 | 5.1492 | 4.8593 |
