# Supplementary material for: Conspicuous colours reduce predation rates in fossorial uropeltid snakes
Source: PeerJ. 2019 Aug 14;7:e7508. doi: 10.7717/peerj.7508 (PMC6698130; doi:10.7717/peerj.7508)
Supplement: Supplemental Information 3 [file peerj-07-7508-s003.docx]

**Supplemental Table S2.**

**Table S2:** Summary and comparison of different models from the GLMM analyses for the field and the captive bird experiments.

| **Model** | | | | LogLik | AIC | ΔAIC |
| --- | --- | --- | --- | --- | --- | --- |
| **Dependant variable** | **Fixed effects** | | **Random effects** |  |  |  |
| **Field Experiment** | | | | | | |
| Attacks | model phenotype (brown, black, yellow, red & novel colour) | transect/sub-transect, model sequence, batch | | -184.2 | 388.4 | 0 |
| Attacks | Null | transect/sub-transect, model sequence, batch | | -196.4 | 404.8 | 16.4 |
