# Supplementary figures and images for: Conspicuous colours reduce predation rates in fossorial uropeltid snakes
Source: PeerJ. 2019 Aug 14;7:e7508. doi: 10.7717/peerj.7508 (PMC6698130; doi:10.7717/peerj.7508)

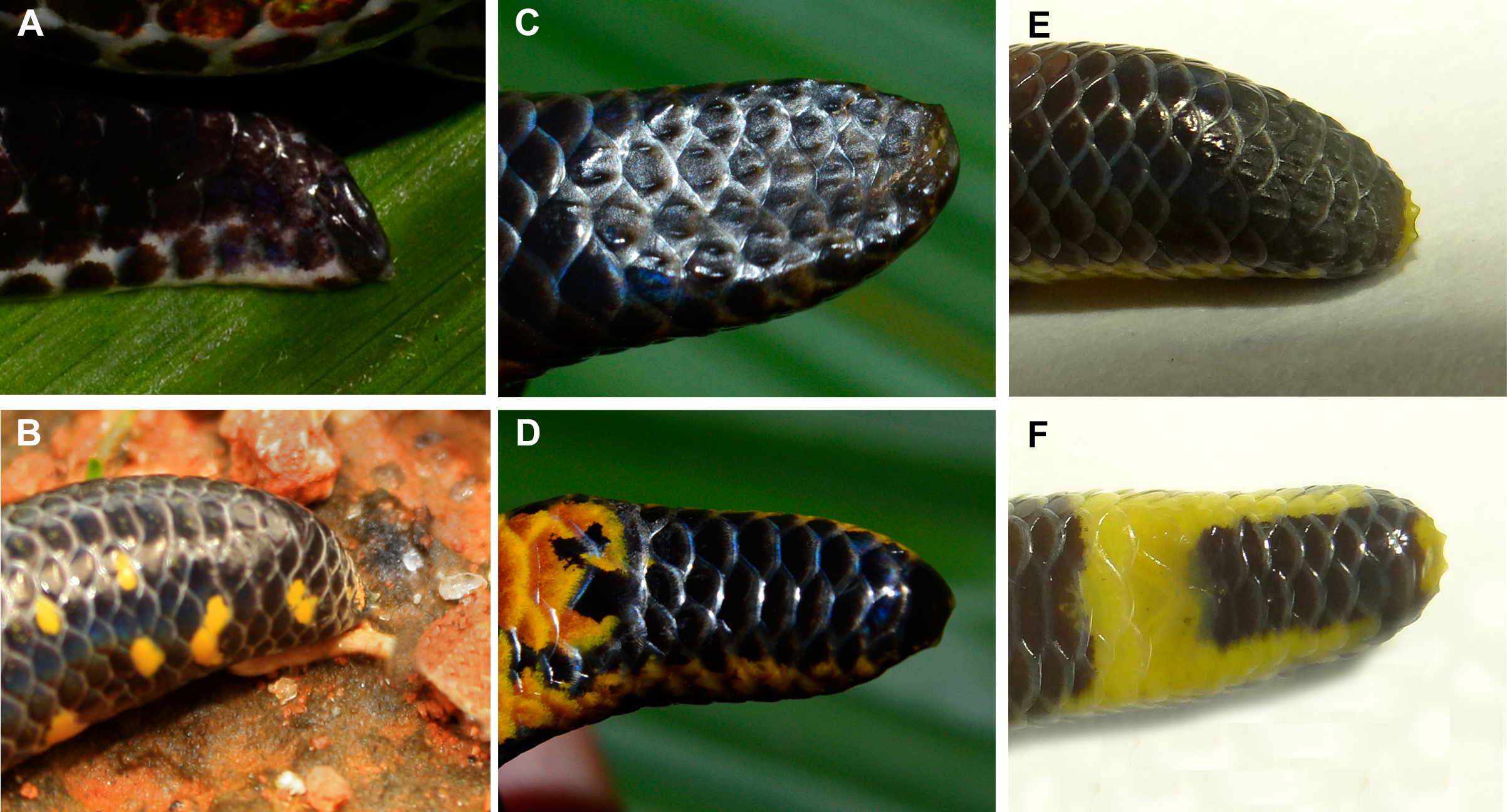

Supplement: Supplemental Information 4 — (A) Lateral view of the tail of Melanophidium punctatum. (B) Lateral view of the tail of Uropeltis liura. (C) and (D) Dorsal and ventral view of the tail of Uropeltis sp. from BBTC tea plantations. (E) and (F) Dorsal and ventral view of the tail of U. cf. ellioti. Photo credit: (A), (C), (D) Umesh P. K. and (B), (E), (F) Vivek Philip Cyriac. [file peerj-07-7508-s004.png]

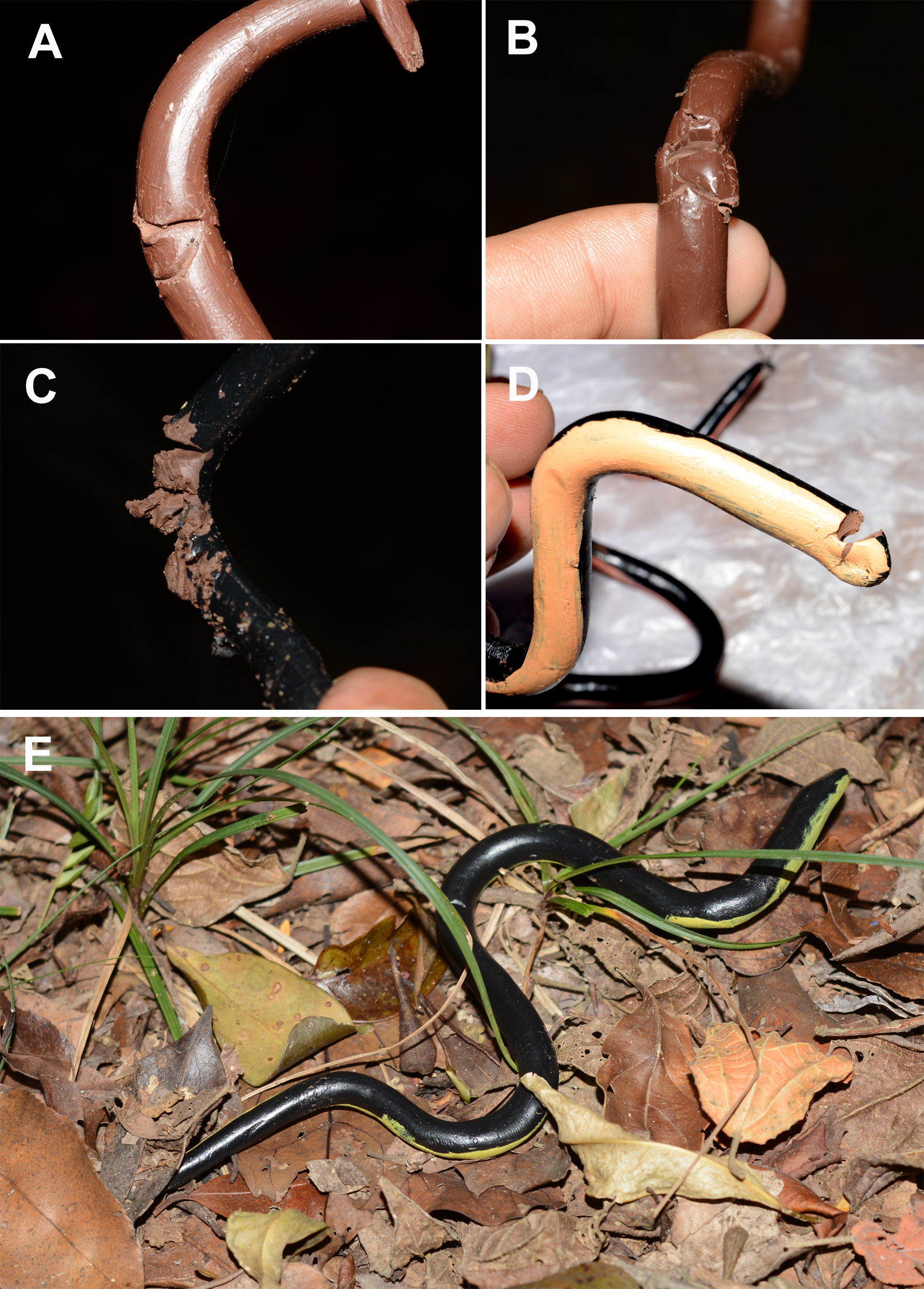

Supplement: Supplemental Information 5 — (A) ‘V’ shaped beak mark of a bird on the clay model. (B) ‘U’ shaped beak mark of a bird on the brown clay model. (C) Multiple peck marks by birds on the black clack model. (D) Peck mark on the tail of a novel coloured model. (E) Snake model (yellow) placed on leaf litter background. Photo credit: Vivek Philip Cyriac. [file peerj-07-7508-s005.png]
